# Unusual presentation of pulmonary adenocarcinoma metastases in the mandibular condyle: A case report

**DOI:** 10.1016/j.ijscr.2023.109058

**Published:** 2023-11-16

**Authors:** Francesco Ferragina, Angelo Ruggero Sottile, Maria Giulia Cristofaro

**Affiliations:** Department of Experimental and Clinical Medicine, Unit of Maxillofacial Surgery, “Magna Graecia” University, Viale Europa, 88100 Catanzaro, Italy

**Keywords:** Condylar metastases, TMD disorders, Maxillofacial Surgery, Surgical Oncology

## Abstract

**Introduction:**

Mandibular bone metastases should be suspected in all patients with temporomandibular joint disorder symptoms and lung cancer history. The purpose of this report is to present a case of metastasis to the mandibular condyle following pulmonary adenocarcinoma.

**Case presentation:**

In December 2020, a 71-year-old patient was evaluated by the Department of Maxillofacial Surgery for the presence of a large osteolytic lesion in the left mandibular condyle. There were no changes to the face or occlusion, and mandibular movements were preserved. After surgical removal, histology revealed pulmonary adenocarcinoma metastasis.

**Discussion:**

To date, only 7 cases of condylar metastases are described by lung cancer. This pathology's clinical and radiological features are almost always shaded and not specific.

**Conclusion:**

This study also focuses on rare conditions, such as metastases to the mandibular condyle. It also stresses the importance of a multidisciplinary approach both in the diagnostic and therapeutic process.

## Highlights

• Metastases to the mandibular condyle from lung adenocarcinoma are a rare and little-known cone. They manifest themselves with non-specific disorders and for this reason, they are a pathology of difficult diagnosis.
• The presence of a multidisciplinary team is fundamental: it allows more professionals to exchange ideas and experiences, determining a diagnostic and therapeutic advantage for the patient.
• To date, there are no therapeutic standards for the surgery of mandibular bone metastases. The case presented can therefore be of help.

## 1 Introduction

Lung cancer represents approximately 12 % of worldwide new cancer diagnoses [1]. In the initial stages, it is almost always asymptomatic, and therefore the diagnosis is late, in the advanced stage when the patient has already developed metastases. Indeed, bone metastases are reported in the literature in 65–75 % of patients with advanced-stage lung, breast, prostate, and bladder cancer [2]. Metastases at the level of the facial massif are rare: they account for about 1–8 % of oral cancer [[3], [4], [5], [6]] and in 60–80 % of cases involve the body of the mandible [[7], [8], [9]]. Mandibular condyle involvement is extremely rare. To date, only a few cases have been reported [4] with non-specific symptoms such as pain, swelling, temporomandibular joint dysfunction, lockjaw, and sometimes even pathological fractures [[4], [5], [6], [7],^9^]. Both the rarity of the condylar disease and the absence of specific signs and symptoms (both clinical and radiological) generally lead to a delay in diagnosis and treatment, thus worsening the prognosis of the disease. Below we present a case of a patient with metastases in the left mandibular condyle originating from lung cancer that showed slight and temporary common temporomandibular joint disorder (TMJ)-like symptoms. The work has been reported in line with the SCARE criteria [10].

## 2 Case presentation

**Fig. 1:**
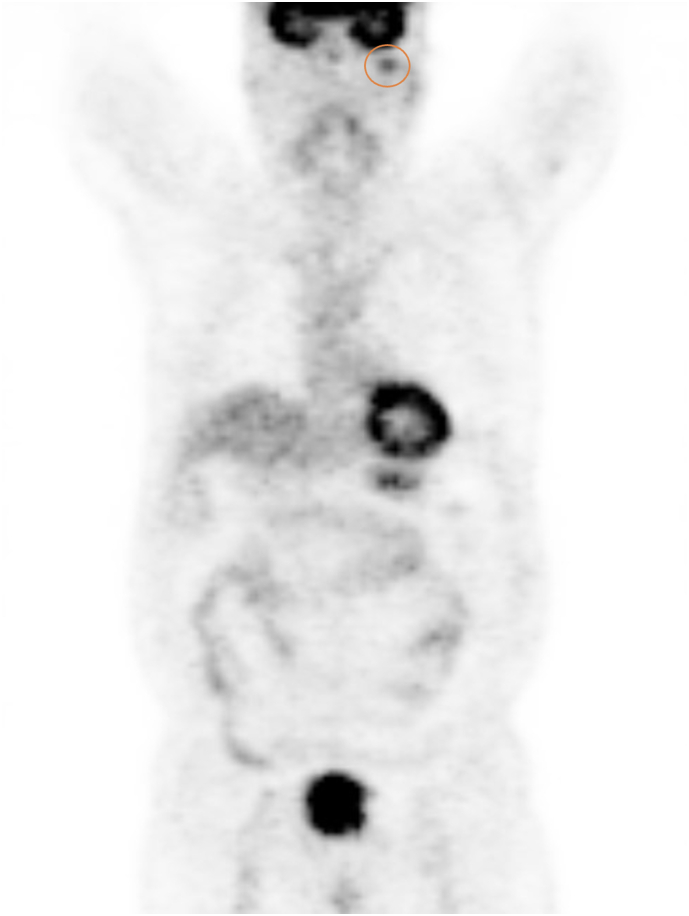
PET examination showing the presence of a focal impregnation area of the radiopharmaceutical of dubious nature (highlighted in the circle) in the left mandibular condyle.

**Fig. 2:**
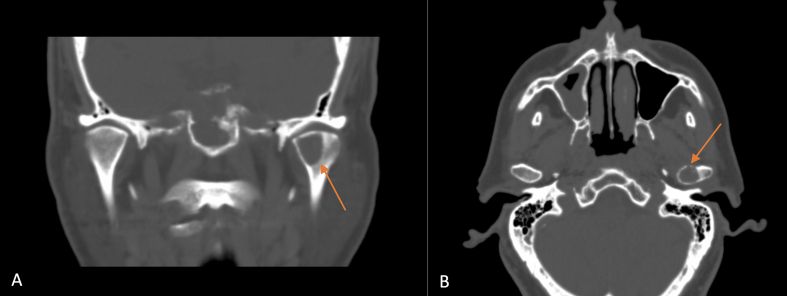
Coronal (A) and Axial (B) CT images of osteolytic lesion in the left mandibular condyle.

**Fig. 3:**
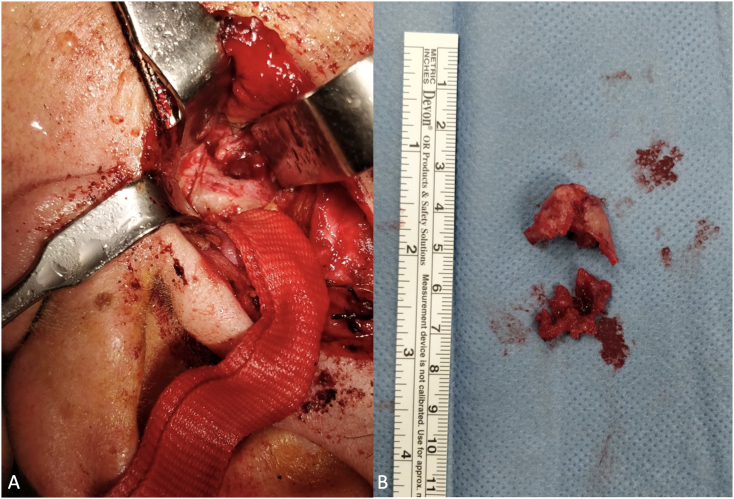
(A) Intraoperative vision of condilectomy performed by piezo-surgery; (B) Intraoperative vision of removed left mandibular condyle with part of surrounding soft tissues (pterygoid muscles).

A 71-year-old white man presented to the Maxillofacial Unit in December 2020 for a specialist surgical consultation. The patient's medical history was positive for lung adenocarcinoma; He had undergone right upper lung lobectomy surgery and mediastinal lymph node sampling in 2019. He also underwent four cycles of adjuvant chemotherapy in 2019. During the Positron Emission Tomography (PET) follow-up, the presence of an osteolytic area in the left condyle was highlighted in December 2020 (Fig. 1). This lesion featured focal impregnation of the radiopharmaceutical of dubious significance, with a maximum SUV (Standardized Uptake Value) of 3.6. There were no facial or occlusion alterations on inspection, and the mandibular movements were preserved. Furthermore, the patient did not report pain or sensorineural alterations. Computed tomography (CT) showed the presence of a lesion of the left mandibular condyle with partial erosion of the cortical bone (Fig. 2 A–B). Magnetic resonance imaging (MRI) also showed a subtle alteration in the surrounding muscles. A multidisciplinary team was created with maxillofacial surgeons, oncologists, and radiotherapists. It was decided to proceed with tumorectomy and subsequent adjuvant radiotherapy. In January 2021 he underwent a left condilectomy under general anesthesia (Fig. 3 A–B). Surgery was extensive: 1.5–2 cm removal from seemingly healthy margins. Then a condylar implant was inserted to restore function. Histopathological analysis revealed bone localization of lung adenocarcinoma; and positive immunophenotype for TTF1 and CK7. The surgical scar healed well and a follow-up CT scan in 3 months displayed no evidence of tumor recurrence. In May 2021, he underwent Stereotactic Body Radiation Therapy every other day for a total of 3000 cGy. In December 2021 RM showed the presence of an expansive solid mass (26 × 23 × 35 mm – AP × LL × SC) in correspondence to the residual condylar region. It presented a homogeneously intermediate signal intensity in both T1w and T2w; a modest alteration of the signal in the neighboring soft tissues was associated (absence of clear signs of the infiltration of the masseter and lateral pterygoid muscles). The patient underwent chemotherapy. The patient, 32 months after surgery, is free from disease.

## 3 Discussion

Metastases are the most important cause of cancer-related morbidity and mortality. Literature suggests that jaws are not a common site of metastasis and condylar metastases are even rarer. These account for 5.7 % of all maxillary metastases [8] and only a few cases have been reported in the literature [9]. To date, only 7 cases of condylar metastases are described. In most cases (22.5 %) they originate from occult pulmonary adenocarcinoma at the metastatic stage [[11], [12], [13]]. Other primary cancers are breast, kidney, liver, prostate, and bladder [3,[14], [15], [16], [17], [18]]. This low rate of condylar localization is linked to two pathophysiological hypotheses [4,[12], [13], [14],^19^]: (1) TMJ has a lower amount of red marrow than other bones of the body; (2) the condyle has a separate blood supply to the mandibular body, so the likelihood of metastases hitting the condyle is lower. Recent literature suggests that metastases mainly affect bones rich in red marrow; at the endothelium level, there is a microenvironment ideal for their proliferation. This theory explains why bone metastases are more frequent in the vertebrae, ribs, pelvis, and extremities of long bones; on the contrary, they are infrequent in the hands, feet, and mandibular condyles. The reason for this selectivity is little known, but it is thought to be due to the concomitant presence of some factors at the level of trabeculae. They are: higher rates of bone turnover; abundant vascularization; microenvironment made of adipocytes, fibroblasts, chemokines, reticulocytes, chondrocytes, endothelial cells, pericytes, hematopoietic and mesenchymal stem cells; bone matrix (made of inorganic salts and organic matrix) acts as structural support for both bone cells and metastases. All this therefore contributes to the seeding of cancer cells and their growth [20].

The clinical presentation of this pathology, as in the case report presented, is shaded and non-specific. Swelling, pain, click joint, and lockjaw are symptoms common to all joint pathologies, especially in patients with comorbidities or known dysfunctional pathologies. Instrumental examinations are also not very specific [4,9,14]. Non-specific symptoms and little knowledge of the pathology (given its rarity) lead to a misdiagnosis and therefore diagnostic delay. In the case presented, the CT and MRI scans reported the presence of an osteolytic lesion; it also highlighted osteoarthrosis processes and inflammation, all confounding factors in the diagnostic process. Surely, the presence of masticatory muscle involvement drove the diagnostic suspect. A possible confounding factor in the present case was the ambiguity of the outcome of the PET examination. The PET has evidenced the presence of osteolytic lesion to condylar level in the absence of an elevated SUV, characterizing a frankly neoplastic lesion. Therefore, a multidisciplinary approach is fundamental in diagnosis: a positive history of pulmonary adenocarcinoma suggests it may have been pulmonary metastasis with rare localization to the mandibular condyle.

## 4 Conclusion

Although metastases are the most important cause of cancer-related morbidity and mortality in the world, lung cancer rarely metastasizes to the maxillary bones. In addition, signs and symptoms associated with the presence of maxillary metastases are nonspecific. These two characteristics make the eventual diagnosis of mandibular metastases, especially condylar, difficult, leading to misdiagnosis. Faced with such conditions, therefore, a multidisciplinary approach is fundamental. It allows you to get to the correct diagnosis faster and then set up a suitable and timely therapy.

## Consent

Written informed consent was obtained from the patient for publication and any accompanying images. A copy of the written consent is available for review by the Editor-in-Chief of this journal on request.

## Ethical approval

The study was conducted following the Declaration of Helsinki; the Magna Graecia University's Ethics Committee approved the study.

## Funding information

This research received no external funding.

## Guarantor

Dr. Cristofaro Maria Giulia.

## CRediT authorship contribution statement

**Francesco Ferragina:** Conceptualization, Data curation, Formal analysis, Funding acquisition, Investigation, Methodology, Project administration, Resources, Software, Visualization, Writing – original draft, Writing – review & editing. **Angelo Ruggero Sottile:** Investigation, Resources, Software, Writing – original draft. **Maria Giulia Cristofaro:** Conceptualization, Data curation, Supervision, Validation, Visualization, Writing – review & editing.

## Conflict of interest

The authors declare that they have no conflict of interest.

## References

[1] Vettori E., Borella A., Costantinides F., Rizzo R., Maglione M. (2023). Mandibular metastasis of pulmonary adenocarcinoma: how unexpected could it be?. Gerodontology.

[2] Dodo M., Kumagai M., Kato Y., Hirakawa H., Koseki T. (2017). Metastasis in the mandibular condyle: a case report. J Med Case Reports.

[3] Hirshberg A., Berger R., Allon I., Kaplan I. (2014 Dec). Metastatic tumors to the jaws and mouth. Head Neck Pathol..

[4] Oliver C., Mouallem G., Dutot-Philipeau N., Longis J., Piot B., Bertin H. (2022 Jun). A case report of condyle metastasis and a review of the literature. J. Stomatol. Oral Maxillofac. Surg..

[5] Kruse A.L., Luebbers H.T., Obwegeser J.A., Edelmann L., Graetz K.W. (2010 Aug). Temporomandibular disorders associated with metastases to the temporomandibular joint: a review of the literature and 3 additional cases. Oral Surg. Oral Med. Oral Pathol. Oral Radiol. Endod..

[6] Johnson C., Read-Fuller A. (2020). Mandibular metastasis from lung adenocarcinoma as the first sign of occult malignancy. Proc. (Bayl. Univ. Med. Cent.).

[7] Baum S.H., Mohr C. (2018 Jun). Metastases from distant primary tumours on the head and neck: clinical manifestation and diagnostics of 91 cases. Oral Maxillofac. Surg..

[8] Qiu Y.T., Yang C., Chen M.J., Qiu W.L. (2013 Apr). Metastatic spread to the mandibular condyle as initial clinical presentation: radiographic diagnosis and surgical experience. J. Oral Maxillofac. Surg..

[9] Freudlsperger C., Kurth R., Werner M.K., Hoffmann J., Reinert S. (2012 Mar). Condylar metastasis from prostatic carcinoma mimicking temporomandibular disorder: a case report. Oral Maxillofac. Surg..

[10] Agha R.A., Franchi T., Sohrab C., Mathew G., Kirwan A., Thomas A. (2020). The SCARE 2020 guideline: updating consensus Surgical Case Report (SCARE) guidelines. Int. J. Surg..

[11] Matsuda S., Yoshimura H., Yoshida H., Umeda Y., Imamura Y., Sano K. (2018 Apr). Mandibular metastasis as the first clinical indication of occult lung adenocarcinoma with multiple metastases: a case report. Medicine (Baltimore).

[12] Gultekin S.E., Senguven B., Isik Gonul I., Okur B., Buettner R. (2016 Oct). Unusual presentation of an adenocarcinoma of the lung metastasizing to the mandible, including molecular analysis and a review of the literature. J. Oral Maxillofac. Surg..

[13] Guarda-Nardini L., Stellini E., Di Fiore A., Manfredini D. (2017). A rare case of misdiagnosed silent lung cancer with solitary metastasis to the temporomandibular joint condyle. J. Oral Facial Pain Headache.

[14] Katsnelson A., Tartakovsky J.V., Miloro M. (2010 Aug). Review of the literature for mandibular metastasis illustrated by a case of lung metastasis to the temporomandibular joint in an HIV-positive patient. J. Oral Maxillofac. Surg..

[15] Cristofaro M.G., Giudice A., Colangeli W., Giudice M. (2011 Jul–Aug). Unique and rare bone metastases from occult primary cancer. Our experience. Ann. Ital. Chir..

[16] Rocha B.A., Paranaíba L.M., Dantas C.D., de Carvalho M.G., de Melo-Filho M.R., Lima L.M., Souto G.R., Horta M.C. (2020). Two rare cases of oral metastasis arising from lung adenocarcinoma and esophageal carcinoma. J Clin Exp Dent.

[17] Park J., Yoon S.M. (2019 Dec). Radiotherapy for mandibular metastases from hepatocellular carcinoma: a single institutional experience. Radiat. Oncol. J..

[18] Gupta N., Khare A. (2020 Apr–Jun). Isolated mandibular metastasis detected on staging 18FDG PET/CT scan in a case of carcinoma urinary bladder. Indian J. Nucl. Med..

[19] Yasar F., Oz G., Dolanmaz D., Akgünlü F. (2006 Sep). Mandibular metastasis in a patient with pulmonary adenocarcinoma. Dentomaxillofac. Radiol..

[20] Fornetti J., Welm A.L., Stewart S.A. (2018 Dec). Understanding the bone in cancer metastasis. J. Bone Miner. Res..

